# Editorial: Drivers of antimicrobial resistance during and after COVID-19 pandemic in low-middle-income countries

**DOI:** 10.3389/fpubh.2024.1521798

**Published:** 2024-12-18

**Authors:** Zia Ul Mustafa, Tauqeer Hussain Mallhi, Yusra Habib Khan, Khezar Hayat, Muhammad Salman

**Affiliations:** ^1^Department of Pharmacy Services, District Headquarter (DHQ) Hospital, Pakpattan, Pakistan; ^2^Discipline of Clinical Pharmacy, School of Pharmaceutical Sciences, Universiti Sains Malaysia, Gelugor, Pulau Pinang, Malaysia; ^3^Department of Clinical Pharmacy, College of Pharmacy, Jouf University, Sakaka, Saudi Arabia; ^4^School of Pharmacy, Faculty of Health and Medical Sciences, Taylors University, Subang Jaya, Selangor, Malaysia; ^5^Institute of Pharmaceutical Sciences, University of Veterinary and Animal Sciences, Lahore, Pakistan; ^6^Institute of Pharmacy, Faculty of Pharmaceutical and Allied Health Sciences, Lahore College for Women University, Lahore, Pakistan

**Keywords:** antibiotics use, antimicrobial resistance (AMR), antimicrobial stewardship (AMS), antimicrobial use, low- and lower-middle-income countries

Antimicrobial resistance (AMR) is among the top 10 global health threats compromising healthcare delivery systems due to associated morbidity, mortality and economic loss (1). It is estimated that 1.14 million deaths (95% uncertainty interval: 1.00–1.28 million) were attributed to bacterial AMR in 2021 (2). Irrational antimicrobial agents' use is one of the key contributors to the development of AMR and the highest mortality and economic burden associated with AMR has been seen in low- and middle-income countries (LMICs) (2, 3).

COVID-19 pandemic is arguably the most devastating health crisis faced by humanity since the influenza pandemic in 1918 (4, 5). The disease has claimed the life of millions of people since its emergence in late 2019 (6). At the start of pandemic, several therapeutics modalities were used to manage COVID-19 patients (e.g. symptomatic treatment, drug repurposing, antivirals, convalescent plasma etc.) as there was no approved treatment or vaccine for the disease existed that time. In addition to the above-mentioned treatments, inappropriate antibiotics use was rampant amid COVID-19 pandemic, adding to the already prevalent issue of AMR, a pandemic within a pandemic. Consequently, our Research Topic delves into the main drivers of AMR during and after COVID-19 pandemic. This articles' collection can help highlight various factors contributing to AMR, thus helping heath authorities to devise further strategies to counter the threat of AMR. We are grateful to all the contributors to our Research Topic. This Research Topic includes a total of eight articles. (1) Tran-Quang et al. revealed high rates of AMR among *Streptococcus pneumoniae* strains isolated from Vietnamese children against common antibiotics like penicillin, erythromycin, and clarithromycin, which is concerning. (2) Chizimu et al. reported low implementation of antimicrobial stewardship (AMS) programs according to the WHO key components in eight health facilities across the five provinces of Zambia. They identified several factors associated with the low implementation of AMS programs e.g. poor functionality of Drugs and Therapeutics Committees, non-performance of AMS actions, lack of education and training, poor reporting of AMS feedback, financial constraints, and limited leadership commitment. (3) Alemkere et al. explored the etiquettes of the antibiotic decision-making process of surgical prophylaxis at a specialized hospital in Addis Ababa, Ethiopia. They observed that deeply ingrained customs hamper evidence-based surgical antibiotic prophylaxis decisions, leading to suboptimal practices. Therefore, there is a dire need to improve current practices under AMS program to optimize surgical antibiotic prophylaxis and mitigate surgical site infections' risk. (4) Rafi et al. assessed the availability of 103 essential antibiotics (Access = 51, Watch = 29, Reserve category = 6 and anti-tuberculosis drugs = 17) in the second largest city of Pakistan (Lahore). They revealed that many essential antibiotics (20%−30%) were not available in both public and private sectors facilities, a serious issue that needs to be addressed by engaging stakeholders and government bodies to overcome supply chain barriers. (5) Song et al. reported that multidrug resistant-tuberculosis remained a significant global health concern, particularly in regions with lower socio-demographic indexes, hence the need for comprehensive, multifaceted tuberculosis management approaches to address this issue. (6) Our Research Topic also included protocol of a community-based survey in different villages of Tigiria block of Cuttack District, Odisha, India, to assess knowledge, attitude and practices of antibiotics use and AMR. Finding from such a survey can provide insights on the awareness and practices of antibiotics use among rural population, consequently helping health regulators to address suggested areas of improvement. Aside from the aforementioned articles, our Research Topic also featured two studies from upper-middle-income countries (China and Brazil). (7) de Bastiani et al. evaluated bacterial profiles and various antimicrobial resistance genes from intensive care units of 41 hospitals across 16 states of Brazil. They reported a wide variety of microbial populations with a recurring presence of bacteria commonly involved in hospital-acquired infections (*Streptococcus* spp., *Corynebacterium* spp., *Staphylococcus* spp., *Bacillus* spp., *Acinetobacter* spp., and bacteria from the Flavobacteriaceae family) among most of the hospitals. They also found that 21% of sanitizing solutions collected showed viable bacterial growth with AMR genes detected. These findings emphasize the adoption of effective surveillance programs to improve infection prevention practices. (8) Li et al. reported decline in AMR prevalence following the implementation of AMS policy at a tertiary hospital in China. However, they did report a significant increase in penicillin-resistant *Streptococcus pneumoniae*, carbapenem-resistant *Escherichia coli* and carbapenem-resistant *Klebsiella pneumoniae* which calls for continuous monitoring of AMR patterns.

Overall, the article featured in this Research Topic underscores the crucial importance of judicious use of antimicrobial agents and overcoming the barriers to implementing AMS to combat AMR. We are deeply grateful to all the authors for their valuable contributions to this critical topic. Additionally, we extend our thanks to the reviewers for dedicating their time to evaluate and enhance the research articles. We hope the content published in our Research Topic significantly contributes to global efforts in tackling antimicrobial resistance.
